# CBME framework to promote transition to senior

**DOI:** 10.36834/cmej.69259

**Published:** 2020-08-06

**Authors:** Amy Acker, Emily Hawksby, Kirk Leifso

**Affiliations:** 1 Department of Pediatrics, Queen’s University, Ontario, Canada

## Implication Statement

Residency programs across Canada are transitioning to Competence by Design (CBD). Our innovative framework improves the traditional transition to independent senior overnight call process by adding workplace-based (WBA) assessments. Our process ensures that faculty and residents have a shared understanding of what competencies need to be demonstrated before residents can work independently as the in-house senior resident overnight. This protocol is worth exploring; initial perceptions suggest an increase in resident confidence while on call and improved faculty comfort when paired with these senior residents. We believe that this in turn will be reflected in enhanced patient care.

## Déclaration des répercussions

Les programmes de résidence partout au Canada sont en cours de transition vers la Compétence par conception (CPC). Notre cadre novateur favorise la transition traditionnelle vers les gardes de nuit par des résidents senioren ajoutant des évaluations en milieu de travail (WBA). Notre processus assure que le corps professoral et les résidents ont une compréhension commune des compétences dont les résidents doivent faire preuve avant qu’ils puissent travailler indépendamment comme résident senior de garde sur place la nuit. Ce protocole mérite d’être exploré; les premières impressions indiquent que la confiance des résidents augmente lorsqu’ils sont de garde et que le corps professoral est plus à l’aise lorsqu’il est jumelé à ces résidents seniors. Nous croyons qu’en retour, ceci rehaussera les soins des patients.

## Introduction

As Canadian residency programs implement Competence by Design (CBD), we report an innovative framework that integrates our traditional way of transitioning residents to senior level independent overnight call with a workplace-based assessment (WBA) process. At our institution, senior pediatric residents take overnight call in the hospital and are paired with staff pediatricians who are out-of-house. In the past, the decision making around this transition has been informal and based on faculty discussions, “hall-way chats/recommendations,” and overall perceptions of resident performance. Residents completed buddied call shifts; however, there was no formal assessment of their performance. As we implement competence-based assessment and promotion, our department felt it was imperative that all decisions regarding progression were based upon clearly defined expectations.

With the new framework in place, before a senior resident can take independent call, they must undergo a transition process that includes three core components: 1) buddied overnight shifts paired with a more senior resident, 2) assessment rubrics to evaluate on-call competencies, and 3) a clear expectations document delineating the core competencies to be demonstrated before a resident can be “unbuddied” (see eSupplement).

## Innovation

Senior call “involves not only demonstrating competency…but also involves the balancing of competing on-call demands and adjusting practices in the face of contextual challenges.”^1^ Our framework meets these requirements. Mapping Entrustable Professional Activities (EPAs) to the graded independence of the Buddy system allows residents to be assessed on the unique requirements for this role in a formally structured and consistent manner.

**Figure 1:**
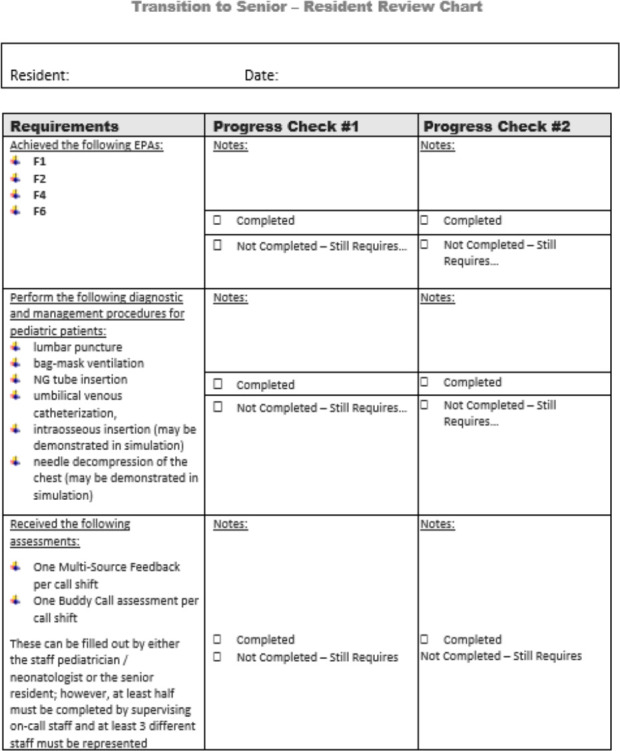
Resident transition to senior review chart

Our residency program continues to use “buddied-call” along with WBA data to ensure that the process to transition residents is reliable and consistent. To clarify expectations, a “Criteria for Independent Senior In-Hospital Call” document (eSupplement) outlines the EPAs that must be completed, how many successful Buddy shifts must have occurred, and additional training requirements that must be met before the resident can be “unbuddied.” The EPAs selected reflect the expectation that residents are competent in the assessment of acute/complex pediatric problems, initial steps of neonatal resuscitation, and recognizing an unstable patient before they can take independent call. Residents must demonstrate completion of key procedures that have been highlighted as important for independent overnight call. Progression is reviewed and documented at Competence Committee and reported back monthly to each resident until successful transition occurs (Figure 1).

## Outcomes

For the previous 18 months we have transitioned our residents using this new structured process. On average each resident had 25 buddied call rubrics completed: 75% completed by faculty and 25% by senior residents. An average of 58 EPA assessments have been completed across the 4 EPAs. Successful completion of an EPA requires 10-20 completed assessments with a score of 4 or 5 on a 5-point entrustment scale.

Initial perceptions discussed at Competence Committee and reported from Academic Advisor meetings suggest an increase in resident confidence while on call and improved faculty comfort when supervising call responsibilities for transitioning and transitioned residents. Areas for future research involve expanding this framework to other departments and examining the full impact of evidence-informed transition to independent senior call on residents, faculty and patient care.
